# Tunability of Size and Magnetic Moment of Iron Oxide Nanoparticles Synthesized by Forced Hydrolysis

**DOI:** 10.3390/ma9070554

**Published:** 2016-07-08

**Authors:** Ben Sutens, Tom Swusten, Kuo Zhong, Johanna K. Jochum, Margriet J. Van Bael, Erik V. Van der Eycken, Ward Brullot, Maarten Bloemen, Thierry Verbiest

**Affiliations:** 1 Department of Chemistry, Laboratory for Molecular Electronics and Photonics, KU Leuven, Celestijnenlaan 200D, Box 2425, 3001 Leuven, Belgium; ben.sutens@chem.kuleuven.be (B.S.); tom.swusten@chem.kuleuven.be (T.S.); kuo.zhong@kuleuven.be (K.Z.); ward.brullot@kuleuven.be (W.B.); maarten.bloemen@kuleuven.be (M.B.); 2 Department of Physics and Astronomy, Laboratory of Solid State Physics and Magnetism, KU Leuven, Celestijnenlaan 200D, 3001 Leuven, Belgium; johanna.jochum@kuleuven.be (J.K.J.); margriet.vanbael@kuleuven.be (M.J.V.B.); 3 Department of Chemistry, Laboratory for Organic & Microwave-Assisted Chemistry, KU Leuven, Celestijnenlaan 200F, 3001 Leuven, Belgium; erik.vandereycken@kuleuven.be

**Keywords:** iron oxide, nanoparticle, biocompatible, forced hydrolysis

## Abstract

To utilize iron oxide nanoparticles in biomedical applications, a sufficient magnetic moment is crucial. Since this magnetic moment is directly proportional to the size of the superparamagnetic nanoparticles, synthesis methods of superparamagnetic iron oxide nanoparticles with tunable size are desirable. However, most existing protocols are plagued by several drawbacks. Presented here is a one-pot synthesis method resulting in monodisperse superparamagnetic iron oxide nanoparticles with a controllable size and magnetic moment using cost-effective reagents. The obtained nanoparticles were thoroughly characterized by transmission electron microscopy (TEM), X-ray diffraction (XRD) and Fourier transform infrared (FT-IR) measurements. Furthermore, the influence of the size on the magnetic moment of the nanoparticles is analyzed by superconducting quantum interference device (SQUID) magnetometry. To emphasize the potential use in biomedical applications, magnetic heating experiments were performed.

## 1. Introduction

Iron oxide nanoparticles (IONP) are used in multiple biomedical applications, ranging from drug delivery to magnetic resonance imaging (MRI) contrast agent, bio-sensing and hyperthermia treatments [1,2,3,4,5,6,7,8,9,10]. Besides the requirement of biocompatibility [11], a sufficiently large magnetic moment is necessary for efficient functioning. Because the magnetic moment is directly proportional to the size of the IONP [12], control over size and thus magnetization of biocompatible IONP is needed. Superparamagnetic iron oxide nanoparticles (SPION) are often considered as a potential candidate, since their biocompatibility has already been proven. Furthermore, they offer a high magnetic moment due to their superparamagnetism and as a result do not contain any residual paramagnetism after removal of the external magnetic field [12]. Apart from applications in biomedical sciences, iron oxide nanoparticles can be used in ferrofluids, Faraday isolators, magnetization-induced second harmonic generation, and magneto-optical materials [13,14,15,16,17,18,19]. Common methods to make such particles are co-precipitation with sodium hydroxide or ammonia [20], microemulsion [21], hydrothermal synthesis or thermal decomposition in organic solvents [22,23]. However, most synthesis methods which are able to produce monodisperse magnetite nanoparticles with controllable sizes up to 20 nm are plagued by several drawbacks.

A frequently used synthesis method is the thermal decomposition of iron-oleate with oleic acid as capping agent, described by Park et al. [23]. Highly uniform and monodisperse nanoparticles are synthesized up to 22 nm. A similar method is described by Chen et al. where iron fatty acid complexes are decomposed in various organic solvents [24]. However, the need for very high and controlled temperatures (320–360 °C) remains a significant disadvantage. Sun et al. also made high-quality monodisperse magnetite nanocrystals by thermal decomposition of iron(III) acetylacetonate with 1,2-hexadecanediol in the presence of oleic acid and oleylamine, developing NP with a tunable size from 3 to 20 nm by seed-mediated growth [25]. This often leads to multiple time-consuming growth steps. Another frequently used effective method is the decomposition of iron pentacarbonyl, described by Hyeon et al. [26]. However, iron pentacarbonyl is very expensive and, like its by-products, toxic.

In this study, a large-scale one-pot synthesis is presented which is performed at relatively low temperature using cheap reagents yielding monodisperse, biocompatible magnetite nanoparticles with controllable sizes ranging from 7.6 to 19.0 nm with magnetizations up to 66 emu/g. Alkylamines are used in combination with a forced hydrolysis synthesis, contrary to previously published synthesis methods where they are utilized in thermal decomposition, hydrothermal synthesis or coprecipitation synthesis [24,27,28,29,30]. The synthesis is followed by a ligand exchange to convert the hydrophobic outer layer hydrophilic by using siloxane chemistry. The obtained superparamagnetic nanoparticles are thoroughly characterized using transmission electron microscopy and X-ray powder diffraction, as well as infrared spectroscopy to confirm a successful ligand exchange. Furthermore, a superconducting quantum interference device (SQUID) based magnetometer is used to determine the magnetization, followed by magnetic heating experiments on a self-built setup. Obtained results indicate great potential in biomedical applications, as well as in Faraday rotation.

## 2. Results and Discussion

**Figure 1:**
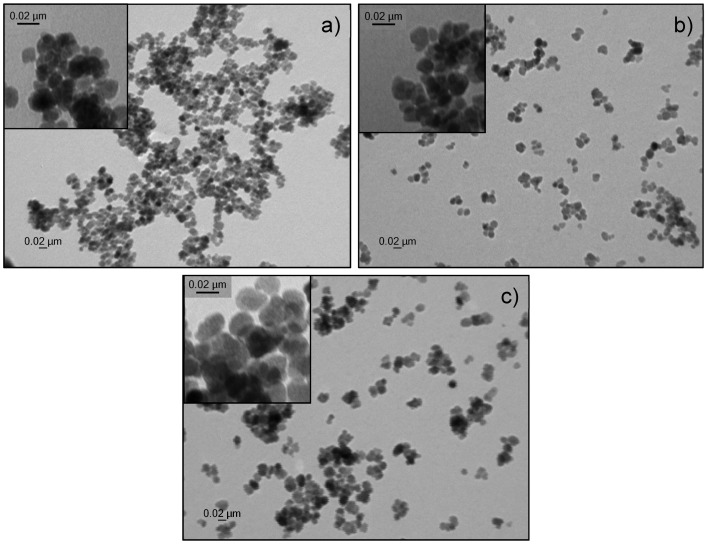
Transmission electron microscopy image of n-octylamine-coated iron oxide nanoparticles. A spherical shape is observed with a size of (**a**) 13.1 ± 3.9 nm (**b**) 16.2 ± 3.1 nm and (**c**) 19.0 ± 3.9 nm.

Control over size and morphology of iron oxide nanoparticles is crucial nowadays for their application in various domains. One of the frequently applied methods to control the size of nanoparticles is to vary the surfactant/precursor ratio. In this study, we present a straightforward one-pot synthesis to create iron oxide nanoparticles with tunable sizes, based on a forced hydrolysis method described by Gangopadhyay et al. [31]. To analyze the role of the surfactant, various amounts of n-octylamine were tested (Table 1). In general the iron precursor, in this case ferric chloride, is added dropwise to a mixture of ethylene glycol, functioning as solvent and reductant, and n-octylamine, which serves as surfactant, at 150 °C. Afterwards, the mixture is heated up to 180 °C and refluxed for 24 h. Above-described method offers several advantages since no extremely high temperatures are needed as the reaction takes place at 180 °C. Also no toxic reagents are used, nor are there hazardous by-products formed. Furthermore, this one-pot synthesis only takes 24 h, as no multiple time-consuming growth steps are needed as opposed to seed-mediated growth of nanoparticles. To determine the size of the obtained n-octylamine-coated nanoparticles, multiple transmission electron microscopy (TEM) images (Figure 1) were taken for each sample, analyzed using ImageJ and fitted with a Gaussian function. This program allows us to count the number of pixels of each individual particle and calculates its size based on the amount of pixels in the scale bar. A clear size reduction is observed due to the increase of the surfactant/precursor ratio, ranging from 7.6 up to 19.0 nm.

**Figure 2:**
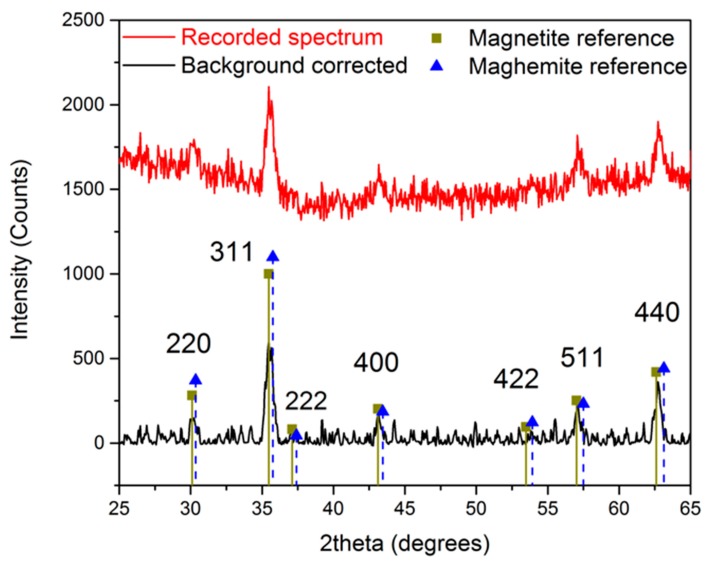
X-ray powder diffraction spectrum of n-octylamine-coated iron oxide nanoparticles.

The crystal structure of the obtained n-octylamine-coated iron oxide nanoparticles is determined by measuring an X-ray powder diffraction spectrum, shown in Figure 2. A reference spectrum of magnetite and maghemite is included to compare the obtained results. Although a close resemblance is observed with the magnetite reference, the exact composition cannot be determined since the difference between maghemite and magnetite is very subtle in XRD powder diffraction. However, we can state with a high degree of certainty that the developed crystals consist of magnetite, maghemite, or a mixture of both. 

**Figure 3:**
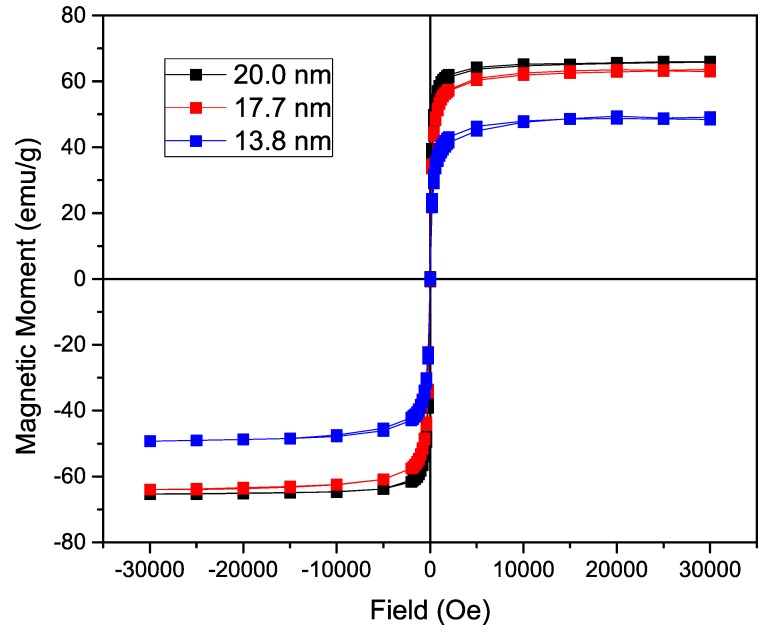
Magnetization of the nanoparticles coated with n-octylamine showing a hysteresis curve with no coercivity or magnetic remanence. A significant increase in saturation magnetization is observed directly proportional to the size of the iron oxide nanoparticles (IONP).

To analyze the influence of the size of the nanoparticle on the magnetization, the n-octylamine coated nanoparticles’ magnetization was determined in a SQUID-based magnetometer. The acquired curves can be found in Figure 3. Using a varying field from 30,000 to −30,000 Oe, a hysteresis curve is formed with a saturation magnetization of 66.0 emu/g for the 19.0 nm IONP, 63.5 emu/g for the 16.2 nm ones and 48.5 emu/g for the 13.1 nm nanoparticles. As expected, while increasing the size of iron oxide nanoparticle core, a significant increase in magnetization is observed due to the lower surface to volume ratio in larger particles. The fact that the magnetization of the 19.0 nm IONP is only slightly higher than the magnetization of the 16.2 nm IONP, can probably be attributed to a different maghemite/magnetite ratio. A Langevin function is used to estimate the magnetic core diameter, assuming non-interacting particles and uniform size. By using the density of magnetite (5.15 g/cm^3^) and the experimentally obtained saturation magnetization (66.0 emu/g, 63.5 emu/g and 48.5 emu/g), following sizes are calculated: 13.66 nm, 13.02 nm and 12.91 nm respectively. Only a small increase in magnetic core size is observed, contrary to the size deducted from the TEM images, due to an increasing large layer of non-magnetic material. Furthermore, the Langevin function assumes non-interacting particles which is not the case and leads to deviations. However, the magnetization remains tunable and comparable to previously reported values [1,32].

Since the magnetic moment scales with the size of the nanoparticles and reaches 66.0 emu/g for 19.0 nm nanoparticles, they can be used in biomedical applications such as hyperthermia. However, to utilize these nanoparticles in biomedical applications, a ligand exchange is crucial to achieve a hydrophilic coating. Frequently used biocompatible coatings for iron oxide nanoparticles are dextran, carboxydextran, chitosan, polyethylene glycol (PEG), poloxamers and polyoxamines [5]. In this paper, we opted for siloxane chemistry where silanol molecules are formed by hydrolyzation of alkoxysilanes. Thiol-ene click chemistry, described by Tucker-Schwartz et al. is used to attach a PEG-chain to a siloxane, resulting in a hydrophilic outer layer [33]. The reason for this is the high density covalent bond formation, high yield, simple reaction conditions and short reaction time. Furthermore, since the by-products of this radical initiator are inert, the final product of the click reaction can be added directly to the functionalization solution. 

**Figure 4:**
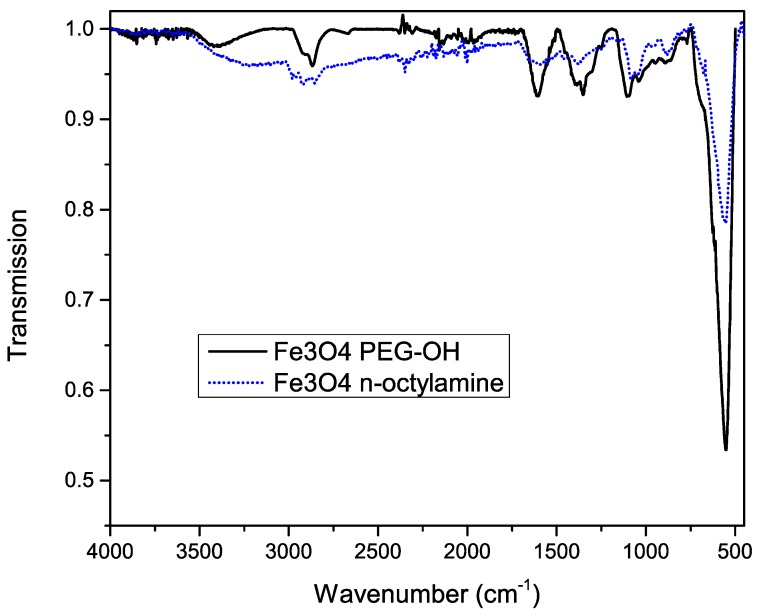
Infrared spectrum of iron oxide nanoparticles before (dotted blue line) and after (solid black line) functionalization with silane-PEG-OH molecules.

To confirm successful functionalization of the nanoparticles’ surface, Fourier transform infrared (FT-IR) spectra were measured before and after the functionalization, shown in Figure 4. A huge peak at 600 cm^−1^ is observed, which is related to the Fe–O bonds, confirming the iron oxide magnetic core. Before functionalization, the asymmetric stretch, the symmetric stretch and scissoring of CH_2_ is observed at 2900, 2850 and 1450 cm^−1^ respectively.

**Figure 5:**
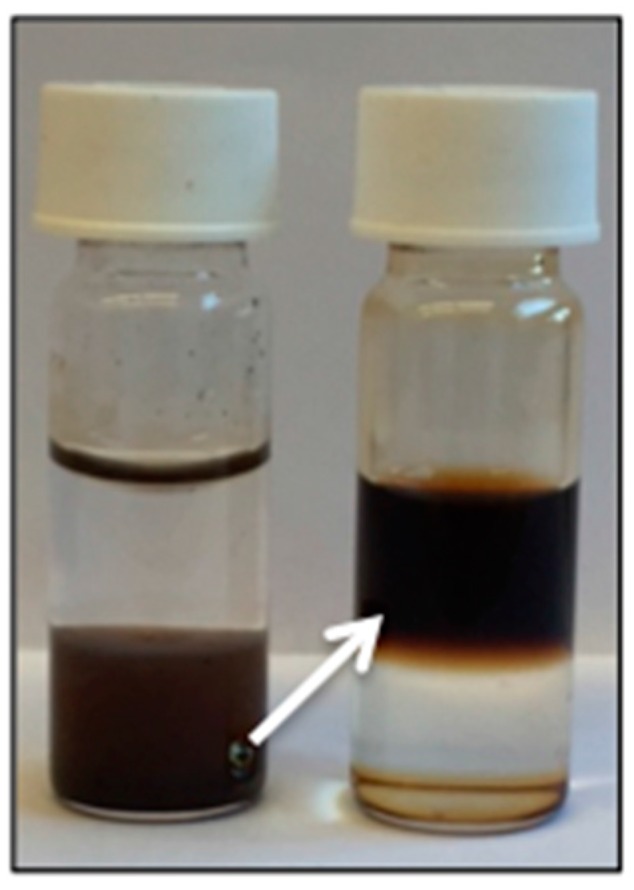
A phase transfer from the organic lower phase to the aqueous upper phase is observed after functionalization with PEG, confirming a successful ligand exchange.

The characteristic broad band at 3300 cm^−1^ after functionalization, which can be attributed to the O–H stretch, confirms the presence of the PEG–OH chains. This is further proved by the broadening of the peak shape at 2860 cm^−1^, corresponding to CH_2_ groups of the PEG chains. Other important features are the 1100, 1060 and 1000 cm^−1^ bands, which are related to the stretching of the Si–O bonds. Since most peaks of octylamine overlap with the peaks corresponding to PEG silane, we can’t exclude residual octylamine. However, the fact that the functionalized particles are water soluble (Figure 5) confirms the successful ligand exchange. 

**Figure 6:**
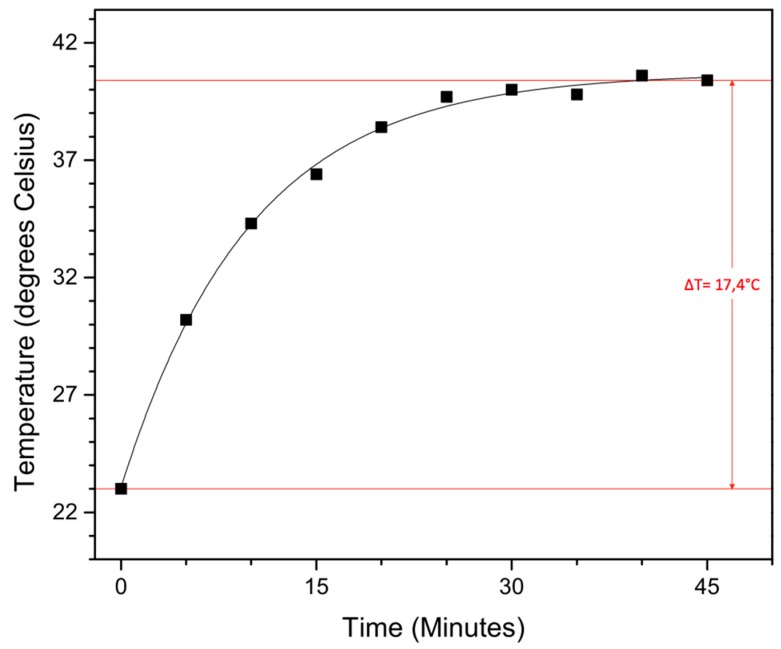
Time-dependent temperature rise from magnetic heating experiments conducted in a 50 Gauss magnetic field (48, 55 kHz) with a 20 nm nanoparticle solution of 0.5 mg/mL in water.

To test their potential in hyperthermia treatment, magnetic heating experiments were carried out on the obtained functionalized 19.0 nm nanoparticles. To function in said application, they should be able to heat malignant cells above 41 °C for 30 min or longer in an applied AC magnetic field, without damaging the surrounding healthy cells. The magnetic field creates an oscillation of the magnetic moment, resulting in a thermal energy release of the magnetic nanoparticles. In our self-built setup, a magnetic field of 50 Gauss (4 kA/m) oscillating at 48.55 kHz (which is not the resonance frequency of our iron oxide nanoparticles, this is situated around 200–250 kHz) is applied to 5 mL of a 0.5 mg/mL nanoparticle solution in water, followed by the determination of the time-dependent temperature rise (Figure 6).

Although the applied magnetic field is slightly lower than previously reported and a fairly low concentration is used, a solvent temperature increase of 17.4 °C is still achieved within 45 min, with a max temperature of 40.4 °C. Although more studies have to be performed, these large superparamagnetic iron oxide nanoparticles with their sufficiently high magnetic moment are potential candidates for hyperthermia treatment.

## 3. Experimental

### 3.1. Materials

Ethylene glycol (99+%), chloroform (for analysis) and n-octylamine (99+%) were purchased at Acros Organics (Geel, Belgium). Ferric chloride (>99%) was ordered at VWR (Leuven, Belgium) and hydroxypolyethoxy (10) allyl ether (98%) was obtained from Polysciences, Inc. (Hirschberg an der Bergstrasse, Germany). (3-mercaptopropyl)trimethoxysilane (95%) was ordered at Sigma Aldrich (Diegem, Belgium), as well as 2,2-dimethoxy-2-phenylacetophenone (99%). Toluene (HPLC grade) was purchased at Fisher Scientific UK (Loughborough, UK).

### 3.2. Synthesis of Nanoparticles

The synthesis of iron oxide nanoparticles was done using a modified forced hydrolysis method described by Gangopadhyay et al. [31]. In general, varying amounts of n-octylamine (Table 1) were added to ethylene glycol (37.5 mL; 0.67 mol) in a three-neck round-bottom flask and heated to 150 °C. Meanwhile, ferric chloride (2.4 g; 0.015 mol) was dissolved in a mixture of ethylene glycol (10 mL; 0.018 mol) and purified water (3.5 mL; 0.19 mol). This solution was added dropwise to the flask. Once the solution was added, the mixture was heated further and refluxed for 24 h at 180 °C. Afterwards, the obtained particles were magnetically precipitated and washed with acetone three times. The particles were then dried in vacuo, resulting in an iron oxide nanoparticle powder with a typical yield of 1 g.

### 3.3. Thiol-Ene Click Chemistry

A functionalized trimethoxysilane was synthesized based on the method published by Tucker-Schwartz et al. [33]. Hydroxypolyethoxy (10) allyl ether (0.249 g; 0.5 mmol) was added to a mixture of (3-mercaptopropyl)trimethoxysilane (0.093 mL; 0.5 mmol) and 2,2-dimethoxy-2-phenylacetophenone (6.4 mg; 0.025 mmol) in 0.5 mL chloroform. The obtained solution was placed in a UV chamber for 1 h on top of a stirring plate.

### 3.4. Nanoparticle Functionalization

The functionalization of the nanoparticles was done by a slightly modified method, based on the protocol described by Bloemen et al. [2]. 50 mg dry iron oxide nanoparticles were added to 50 mL toluene, followed by the addition of 0.025 mL of MilliQ water and 0.5 mL of the prepared siloxane. This solution was then placed in the ultrasonic bath for 5 h at a temperature of 50 °C to ensure maximal dispersion. Afterwards, the particles were placed on a magnet to precipitate the functionalized particles and washed three times with heptane and acetone. The nanoparticles were then dispersed in water with a concentration of 5 mg/mL.

### 3.5. Equipment and Characterization

The UV chamber used in the functionalization is equipped with 3 LEDs (365 nm), with an output power of 200 mW. The ultrasonication bath was a Bransonic Model 5510 sonicator with a capacity of 10 L. An 80 kV Zeiss EM-900 with 300 mesh Formvar coated copper grids was used to perform transmission electron microscopy measurements. Distribution data based on the TEM images were conducted by ImageJ. The Fourier transform infrared spectra were obtained by using a Bruker Alpha FT-IR spectrometer equipped with a Platinum ATR module. Powder X-ray diffraction patterns were recorded using a Bruker D8 Advance X-ray powder diffractometer with CuKα radiation (λ = 1.5406 Å). Magnetic properties of the nanoparticles were characterized by a superconducting quantum interference device (SQUID) magnetometer (LOT-Quantum Design MPMS XL-5, San Diego, CA, USA) in dry state.

## 4. Conclusions

In this work, we reported a straightforward way to tune the size and magnetic moment of superparamagnetic iron oxide nanoparticles. SPION with tunable sizes, ranging from 7.6 to 19.0 nm, were developed via a modified forced hydrolysis method. In this way, the magnetization can be varied from 48.5 up to 66.0 emu/g, making them applicable for biomedical applications. To increase the biocompatibility, a ligand exchange is performed using thiol-ene click and silane chemistry resulting in monodisperse water-soluble IONP. The potential of our synthesized NP is proven in magnetic heating experiments, since a significant solvent temperature increase is achieved within 45 min at fairly low frequencies. 

## Figures and Tables

**Table 1:** Influence of the amount of surfactant on the size of the iron oxide nanoparticle. 10, 15 and 20 mL n-octylamine were tested and compared to the 7.6 nm iron oxide nanoparticles described by Brullot et al. [13].

Surfactant (mL)	Size (nm)
10	19.0 ± 3.9
15	16.2 ± 3.1
20	13.1 ± 3.9
25	7.6 ± 2.1
